# Complex PrEP: the factors requiring consultant-led review of PrEP users

**DOI:** 10.1136/sextrans-2021-055277

**Published:** 2022-02-15

**Authors:** Victoria Tittle, Rebecca Dalton, Diarmuid Nugent, Nicolo Girometti, Gary Whitlock, Alan Mcowan, Sheena McCormack

**Affiliations:** 1 56 Dean Street, HIV, Chelsea and Westminster Hospital NHS Foundation Trust, London, UK; 2 MRC CTU, University College London, London, UK

**Keywords:** pre-exposure prophylaxis, sexual health, health services research

## Abstract

**Introduction:**

HIV-1 pre-exposure prophylaxis (PrEP) has been available in England since March 2020 on the National Health Service using generic emtricitabine and tenofovir disoproxil. 56 Dean Street (56DS) provided PrEP through (1) additional private care from September 2015, estimated to be providing 11% of England’s PrEP in 2019; and (2) the IMPACT trial, as well as monitoring those self-sourcing PrEP. Providing PrEP at scale through a nurse-led service required a safety net for complex individuals. 56DS introduced a consultant-led PrEP outpatient service, the PrEP review clinic, in January 2018 and we report the outcomes of this service.

**Methods:**

We present a retrospective case note review of the PrEP review clinic with descriptive outcomes from 26 January 2018 to 20 December 2019. Reason for referral, demographics, PrEP management and PrEP discontinuations were recorded.

**Results:**

13 980 unique users accessed PrEP from 56DS during the two year evaluation period. 220 individuals were seen in the PrEP review clinic. Majority of patients were referred for renal issues (114 of 220, 51.8%), followed by side effects (59 of 220, 26.8%) and comorbidities (38 of 220, 17.2%). Of those with renal issues, 89 (out of 114, 78.1%) users were referred for an abnormal estimated glomerular filtration rate (eGFR). 35 (out of 114, 30.7%) PrEP users had an eGFR between 45 and 59 mL/min/1.73 m^2^, of whom 2 (5.7%) discontinued PrEP. Majority of users were advised to stop supplements±switch to event-based dosing (24 of 35, 68.6%). Ten PrEP users were referred with an eGFR between 30 and 44 mL/min/1.73 m^2^; 4 (40%) stopped or did not start PrEP and 6 (60%) were asked to stop supplements±switch to event-based dosing.

**Discussion:**

A small proportion of PrEP users have complex PrEP issues. Methods to manage renal dysfunction with PrEP included stopping supplements and switching to event-based dosing. Those with side effects were managed with an array of options, with only modest effectiveness. Other PrEP options are needed to support those with toxicities or intolerances.

## Introduction

HIV-1 pre-exposure prophylaxis (PrEP) was commissioned by the National Health Service England in March 2020 using current market authorisation for generic emtricitabine (FTC) and tenofovir disoproxil (TD).^1^ Since October 2015, PrEP users self-sourced TD/FTC from online pharmacies (facilitated by iwantprepnow and Prepster).^2^ 56 Dean Street (56DS) provided PrEP through additional private care from September 2015 (and are estimated to have provided 11% of England’s PrEP in 2019) and England’s PrEP IMPACT trial from October 2017 to October 2020.^3^

Providing PrEP at scale through a nurse-led service required a safety net for individuals with complex needs, and 56DS introduced a consultant-led PrEP outpatient service in January 2018. In contrast to people living with HIV, who can switch to an alternative drug, there is only one option for PrEP users, TD/FTC, with limited guidance for managing toxicities (online supplemental figure 1). The British HIV Association (BHIVA) recommends use of PrEP with renal dysfunction (estimated glomerular filtration rate (eGFR) less than 60 mL/min/1.73 m^2^) is on a case-by-case basis.^4^

10.1136/sextrans-2021-055277.supp1 Supplementary data

We explore and report our experience from the first 2 years of this consultant-led PrEP review clinic, including reason for referral, management strategies and PrEP discontinuation levels.

## Methods

PrEP users were estimated from the clinic database and duplicates were removed. The PrEP review clinic was weekly, with six appointment slots (30 minutes per an appointment). Referrals ccould be made by any clinic staff member (doctor, nurse, results administrator) by phone, email or in person. There were no referral criteria for the PrEP review clinic, with an open policy for staff to book directly into the clinic if they were concerned, to support nurses and doctors with varying degrees of PrEP experience. Results teams booked PrEP users directly into the PrEP review clinic if the eGFR was <60 mL/min/1.73 m^2^ or the urine protein creatinine ratio (UPCR) was >30 mg/mmol. The algorithm for managing abnormal renal parameters, developed to support clinicians prescribing PrEP in the PrEP IMPACT trial, was used to guide investigations and onward referral for all relevant PrEP users in the PrEP review clinic, regardless of their participation status in the IMPACT trial (online supplemental figure 1). Renal impairment by eGFR was categorised using the National Institute for Health and Care Excellence (NICE) chronic kidney disease categories (table 1).^5^

**Table 1:** PrEP review clinic outcomes and renal PrEP outcomes

Reason for referral to PrEP review clinic	
Reason for referral	Unique PrEP users, n (%)
**Renal**	**114 (51.8)**
Abnormal eGFR and/or high creatinine	89 (40.5)
Proteinuria (>30 mg/mmol and/or ≥1+ protein on urine dipstick)	15 (6.8)
Other renal issues not presenting with abnormal eGFR or proteinuria	10 (4.5)
**Side effects**	59 (26.8)*
**Comorbidities**	**38 (17.2)**
Concerns about safety of PrEP	15 (6.8)
Drug–drug interactions	8 (3.6)
Hepatitis B	8 (3.6)
Bone health	7 (3.2)
**Other**	**9 (4.1)**

Renal eGFR cases: possible cause and management following consultation (n=89)					
Referral eGFR (mL/min/1.73 m^2^)	Protein supplements: to stop these and retest	Alternative explanation, already being monitored	Alternative explanation, no extra monitoring needed	No explanation, further investigated or monitored	Total (n=89)
≥90 (had abnormal eGFR or raised creatinine and normalised on repeat)	2	2	0	1	5
60–89	13	17	4	5	39
45–59	18	8	1	8	35
30–44	3	6	0	1	10

*Out of 59 PrEP users, 47 (76.7%) obtained PrEP on at least one other occasion following their initial PrEP review clinic appointment, 9 (15.3%) had unknown outcomes and 3 (5.1%) have not restarted PrEP due to reasons including an allergic reaction with bullous lesions which recurred on rechallenge and led to discontinuation of PrEP. The regimen for increasing tolerance is provided in online supplemental table 4.

†

eGFR, estimated glomerular filtration rate; PrEP, pre-exposure prophylaxis.

10.1136/sextrans-2021-055277.supp4 Supplementary data

A retrospective clinic note review was undertaken to extract the following details: gender (current and at birth), age in years, ethnicity, sexual orientation, reason for PrEP, reason for referral, management plan and outcome, for PrEP users seen between 26 January 2018 and 20 December 2019. Indication for PrEP was defined as (1) gay, bi and other men having condomless receptive and insertive anal sex with men, (2) transgender women (TGW) having condomless anal sex, and (3) sex with partners from areas of high HIV prevalence or partners living with HIV with a detectable HIV viral load (>200 copies/mL) or within 6 months of starting antiretrovirals. We have used the terminology ‘protein supplements’ where high protein diets, creatinine and protein supplements have been reported. Specific recreational drugs were often not recorded on clinical notes, and the terminology ‘recreational drugs’ refers to any recreational drug use including ‘chems’ (typically crystal methamphetamine, mephedrone, GHB and GBL). Event-based dosing (EBD) refers to IPERGAY dosing regimen (2 pills 2–24 hours prior to sex—1 pill 24 hours later—1 pill 48 hours later).^6^

The clinical outcome of interest was whether PrEP users remained on PrEP, and secondary descriptive outcomes included demographics, reason for referral, summary of management strategies and proportion of PrEP users needing specialist or general practitioner (GP) referral.

## Results

Between 26 January 2018 and 20 December 2019, 13 980 unique users accessed 56DS for PrEP on at least one occasion and 220 (1.6%) of these individuals were seen in the PrEP review service. Demographics of PrEP users seen in the PrEP review clinic were predominantly cis male (216 of 220, 98.2%), of white ethnicity (165 of 220, 75%), born in the UK (117 of 220, 53.2%) and with a median age of 40 years old (IQR 34-50). The reason for seeking PrEP was gay, bi and other men, including trans men having condomless anal sex with men (214 of 220, 97.3%), TGW having condomless anal sex (1 of 220, 0.5%), and partners from areas of high HIV prevalence or partners with detectable HIV viral load (5 of 220, 2.3%). No transgender men were seen in the PrEP review clinic. At the time of the first PrEP review clinic, 168 PrEP users (out of 220, 76.4%) had started TD/FTC. Most were taking PrEP daily (123 of 168, 73.2%). Seventy (out of 89, 78.7%) PrEP users being referred with eGFR issues were on PrEP at their first consultation in the PrEP review clinic, but 45.7% (32 of 70) did not have baseline serum creatinine and eGFR available as they started PrEP prior to attending 56DS.

Table 1 outlines the reasons for referral to the PrEP review clinic, categorised as renal, side effects, comorbidities and other. Online supplemental tables 2 and 3 provide detailed renal outcomes and management for eGFR less than 60 mL/min/1.73 m^2^. Online supplemental table 4 outlines side effect management strategies.

10.1136/sextrans-2021-055277.supp2 Supplementary data

10.1136/sextrans-2021-055277.supp3 Supplementary data

Of the 15 (out of 220, 6.8%) PrEP users being referred with abnormal UPCR, 8 (out of 15, 53.3%) normalised on repeat sampling, 3 (out of 15, 20%) PrEP users continued to be monitored, 3 (out of 15, 20%) switched to EBD and were advised to repeat tests but were lost to follow-up or transferred their care, and 1 (out of 15, 6.7%) had concurrent urethral *Chlamydia trachomatis* infection. Fourteen (93.3%) had an eGFR>60 mL/min/1.73 m^2^ with their abnormal UPCR result, and one user’s eGFR was >60 mL/min/1.73 m^2^ on repeat blood sampling.

Overall, 10 (out of 220, 4.5%) discontinued PrEP due to renal function, side effects or toxicities. Twenty-six (out of 220, 11.8%) PrEP users required discussion with another clinician: 9 (34.6%) with GP, 15 (57.7%) with a specialist team (including hepatology, endocrinology and renal) and 2 (7.7%) required inpatient referrals (to accident and emergency and a local day unit). Four of the nine (44.4%) PrEP users requiring a GP discussion were new referrals to the GP.

Further actions taken for the five PrEP users with an eGFR between 60–89 and no known explanation included referral back to the GP or specialist team (already known to the PrEP user), further renal function investigations, or increased frequency of monitoring at 56DS.

## Discussion

The majority of referrals were for renal issues and side effects (either symptoms or abnormal non-renal blood results). PrEP users with an eGFR 60-90 mL/min/1.73 m^2^ were managed according to the BHIVA guidelines with increased monitoring.^4^ Just over half of PrEP users with an eGFR less than 90 mL/min/1.73 m^2^ were managed by reducing or stopping protein supplements, or less frequently, recreational drugs. They were asked to interrupt protein supplements for a minimum of 2 days prior to a repeat test. Only one user discontinued PrEP in this group as they did not wish to stop protein supplements.

For PrEP users with low stable eGFR of 45–59mL/min/1.73 m^2^, that was not corrected by withholding protein supplements, a risk benefit discussion was undertaken to assist the individuals to determine whether or not they could manage without PrEP. For those whose HIV acquisition risk was substantial, the regimen was tailored to minimise the total exposure to drug, either event-based or four tablets a week (Tuesday, Thursday, Saturday, Sunday or the 2Ts 2Ss aide-memoire developed by the community) with 3–6 monthly renal function monitoring.^7^ This strategy is supported by the IPERGAY open-label extension study observation of a dose–response relationship between the number of pills taken in the preceding 2 months and the slope of eGFR decline, with a small but significant difference in favour of those who had used 15 or less pills compared with more than 15 pills. However, in IPERGAY, the drug was interrupted when eGFR fell below 60 mL/min/1.73 m^2^, so to our knowledge this is the first evidence of using this strategy in PrEP users with eGFR 45–59 mL/min/1.73 m^2^. As this was a retrospective case review, precise pill counts after implementing the strategy and longitudinal eGFR data are not available. When eGFR was less than 45 mL/min/1.73 m^2^ at the first PrEP review clinic, or following a trial of EBD, the risk from the drug outweighed the benefit in almost all cases and in the context of the decline in local HIV incidence during this time.^8^

Pathways and funding for further renal investigation of PrEP users in sexual health settings are currently lacking and sexual health medical records are separate from GPs in England. PrEP users with confirmed low eGFR were usually known to their GP and in some cases under regular follow-up with a specialist for suboptimal control of other conditions such as hypertension or diabetes. With 12% of complex PrEP users requiring discussion with other specialties, pathways are needed for GP input and further investigations, such as nuclear medicine eGFR scans and cystatin-C,. We used the NICE guidelines for chronic kidney disease for renal monitoring, aligning with other medical specialties, and feel BHIVA should consider these in future PrEP guidelines.^4 5^

The frequency of renal abnormalities we observed is similar to the experience in a sexual health centre in Sydney, where 1.9% of PrEP renal monitoring attendances had a reduced eGFR using a slightly higher renal impairment threshold (eGFR less than 65 mL/min/1.73 m^2^).^9^ In Partners PrEP the eGFR decline was −1.59 mL/min/1.73 m^2^ over 18 months, and there was no difference between the placebo and the TD/FTC intervention groups in the proportion of users who developed a 25% drop in renal function.^10^ The iPrEx trial reported a median eGFR decline of −2.9% among their cohort and only 0.1% dropped their creatinine clearance to <60 mL/min.^11^ I Factors associated with declines in renal function are age (over 40 years old), reduced baseline creatinine clearance and comorbidities such as hypertension and diabetes.^11^ Based on these PrEP clinical trial findings, BHIVA guidelines recommend increasing the frequency of renal monitoring from once a year to every 6 months in those over 40 years old, users with hypertension and diabetes mellitus, and those on nephrotoxic medication.^4^ IPERGAY renal data found little difference in mean eGFR decline in EBD users (−0.88 mL/min/1.73 m^2^ per year in the placebo group vs −1.53 mL/min/1.73 m^2^ in the TD/FTC group, with a slope difference of 0.65 mL/min/1.73 m^2^, p=0.27) and no difference in older (>40 years old) users or those with hypertension.

Side effects were the second most common reason for referral to the PrEP review clinic (27%). An attempt to build tolerance by slowly increasing the dose over the course of a week was met with variable success. Individuals with side effects found it easier to evaluate the risks and benefits of PrEP than those with abnormal laboratory parameters and those with severe side effects chose to discontinue. When the relationship to PrEP was unclear, individuals were reminded how to interrupt PrEP safely and advised to take a break for a month at a suitable risk-free time and rechallenge.

While the majority of PrEP users did not require consultant-led review in the PrEP review clinic, there is a group of individuals who may benefit from alternative PrEP options, particularly those with significant renal impairment and severe side effects or toxicities. Tenofovir alafenamide/FTC is now licensed in the USA for men-who-have-sex-with-men PrEP use and cabotegravir efficacy is established.^12 13^

Of note, as HIV incidence continues to decline, the benefit of TD/FTC PrEP has to be weighed against the risks from the drug and the threshold for discontinuing the drug is likely to change. PrEP has undoubtedly had a broader societal impact and it will be important to understand this so that tailored discussions in clinics are in line with messages in the community and vice versa.

### Limitations

The limitations of this evaluation include being a retrospective case note review with variable follow-up, and PrEP interruption and discontinuation may be under-reported. The methodology limits data availability of baseline eGFRs, number of pills taken and longitudinal eGFRs, but is reflective of PrEP management in real-life settings. The population is predominately white and cis male and data cannot be extrapolated to individuals assigned female at birth. The number of PrEP users maybe underestimated as self-sourced online PrEP was not always reported in general sexual health consultations. The number of PrEP users requiring a consultant review will be an underestimate as simple issues will have been managed with consultant advice during routine clinic attendance, but we were unable to obtain these data from routine data coding.

## Conclusion

In the context of 13 980 PrEP users, the number seen in the PrEP review clinic was small, mostly due to renal concerns, and very few PrEP users discontinued PrEP due to adverse events (4.5%) in a real-world setting. Further prospective research to formally evaluate management strategies in PrEP users with eGFR <60 mL/min/1.73 m^2^ is warranted.
