# Supplementary material for: Complex PrEP: the factors requiring consultant-led review of PrEP users
Source: Sex Transm Infect. 2022 Feb 15;98(8):595–8. doi: 10.1136/sextrans-2021-055277 (PMC9685731; doi:10.1136/sextrans-2021-055277)

Supplementary figure 1. IMPACT algorithm for managing abnormal renal parameters (*depending on local pathways & patient preference)

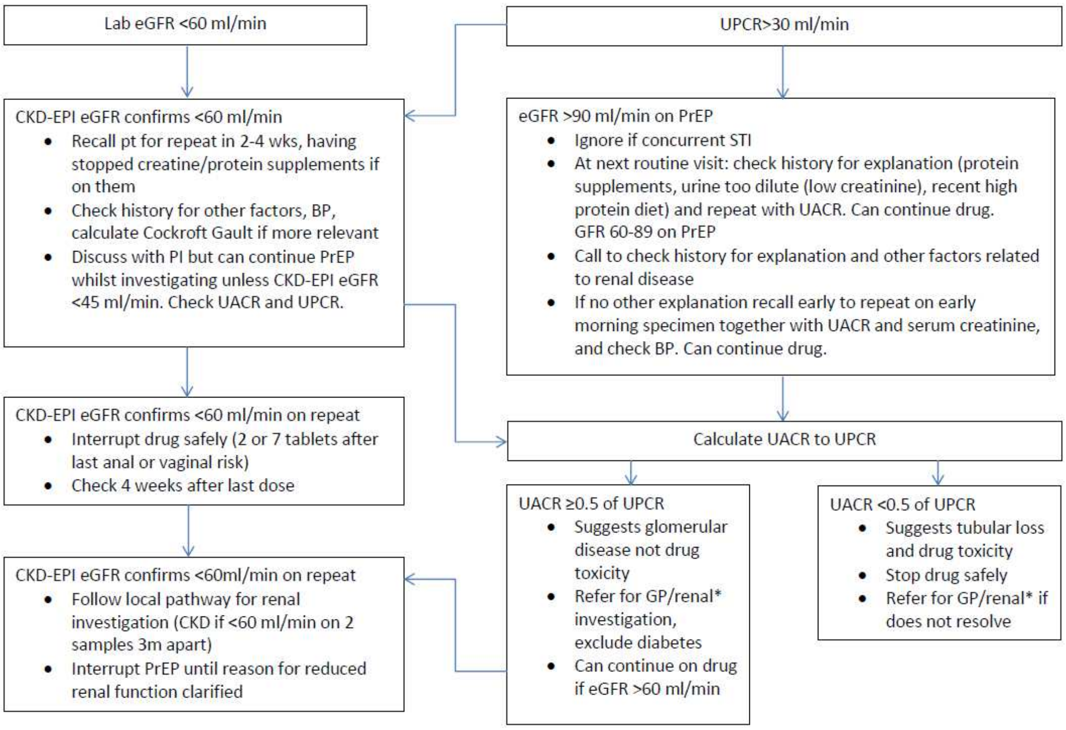

Supplement: Supplementary data [file sextrans-2021-055277supp001.pdf]
