# Supplementary material for: Complex PrEP: the factors requiring consultant-led review of PrEP users
Source: Sex Transm Infect. 2022 Feb 15;98(8):595–8. doi: 10.1136/sextrans-2021-055277 (PMC9685731; doi:10.1136/sextrans-2021-055277)
Supplement: Supplementary data [file sextrans-2021-055277supp004.pdf]

**Supplementary table 4. Summary of side effect management, using 1 or more of the following strategies**
Alternative causes/explanations from history/investigations
Investigation into alternative explanations
Reassurance
Offered allergy clinic review
Stop prep (including bullous allergic reaction, abnormal blood results)
Supportive therapies (anti-histamines, anti-emetics, paracetamol, probiotics)
Slowly build up tolerance to TDF/FTC (take 1/4 pill, ½ pill, whole pill over the course of 3-7 days whilst no risk of HIV)
Switch to EBD
Switch to daily dosing
Switch PrEP brand
Take at different times of the day
Take with food
Trial PrEP break
