# Supplementary material for: Complex PrEP: the factors requiring consultant-led review of PrEP users
Source: Sex Transm Infect. 2022 Feb 15;98(8):595–8. doi: 10.1136/sextrans-2021-055277 (PMC9685731; doi:10.1136/sextrans-2021-055277)
Supplement: Supplementary data [file sextrans-2021-055277supp002.pdf]

**Supplementary table 2. Management plan for PrEP users with referral eGFR 45-59 (ml/min/1.73m^2) (n=35)**								
**Management plan**		**Total number of PrEP users (n)**	**Outcomes:**			**Follow-up eGFR (by CKD category) at median days of =74 days**		
			**Started/ Continued with event-based dosing**	**Started/ Continued with daily dosing**	**Discontinued /decided not to start**	**Improved: eGFR increased >59 (ml/min/1.73m^2)**	**Stable: eGFR remained between 45-59 (ml/min/1.73m^2)**	**Reduction: eGFR reduced to 30-44 (ml/min/1.73m^2)**
Known renal history, eGFR already being monitored. Risk benefit assessed, switched to event-based dosing and more frequent monitoring		3	3	0	0	0	2	1^{+}
High protein intake/supplements/ recreational drugs	Stop supplements and repeat	14	1	12	1	3	9	1^{++}
	Stop supplements, switch to event-based doing and repeat	10 *	7	1	0	4	4	0
Renal function >59 (ml/min/1.73m^2) when using		4	0	4	0	n/a	n/a	n/a

Cockcroft Gault equation							
Repeated bloods/eGFR were >59 (ml/min/1.73m^2)	3	0	3	0	n/a	n/a	n/a
Reviewed risk benefit and decided not to start	1	0	0	1	n/a	n/a	n/a

Footnotes:

[+] reduced by 5 ml/min/1.73m^2, GP reviewing and monitoring

[++]reduced by 4 ml/min/1.73m^2, advised to return to clinic, but not attended

*2 missing because 1 PrEP user requested GP follow-up and was not seen again in clinic and 1 PrEP user was lost to follow-up
