# Supplementary material for: Complex PrEP: the factors requiring consultant-led review of PrEP users
Source: Sex Transm Infect. 2022 Feb 15;98(8):595–8. doi: 10.1136/sextrans-2021-055277 (PMC9685731; doi:10.1136/sextrans-2021-055277)
Supplement: Supplementary data [file sextrans-2021-055277supp003.pdf]

<table>
<tr><th colspan="10">Supplementary table 3. Management plan for PrEP users with referral eGFR 30-44 (ml/min/1.73m^2) (n=10)</th></tr>
<tr><th colspan="2" rowspan="2">Management plan</th><th rowspan="2">Total number of unique PrEP users
(n)</th><th colspan="3">Outcomes (n)</th><th colspan="4">Follow-up eGFR (by CKD category) at median days of =95days</th></tr>
<tr><th>Started/Continued with event-based dosing</th><th>Started/Continued with daily dosing</th><th>Discontinued/decided not to start</th><th>Improved: eGFR increased >59 (ml/min/1.73m^2)</th><th>Improved: eGFR increased 45-59 (ml/min/1.73m^2)</th><th>GFR remained between 30-44 (ml/min/1.73m^2)</th><th>Reduction: eGFR reduced to <30 (ml/min/1.73m^2)</th></tr>
<tr><td colspan="2">Trial of event-based dosing and repeat eGFR, given risk/benefit</td><td>1</td><td>0</td><td>0</td><td>1</td><td>0</td><td>0</td><td>0</td><td>1*</td></tr>
<tr><td rowspan="2">High protein intake/supplements/
recreational drugs and normalised</td><td>Stop supplements and repeat</td><td>3</td><td>3</td><td>0</td><td>0</td><td>2</td><td>1</td><td>0</td><td>0</td></tr>
<tr><td>Stop supplements, switch to event-based doing and repeat</td><td>3</td><td>3</td><td>0</td><td>0</td><td>2</td><td>1</td><td>0</td><td>0</td></tr>
<tr><td colspan="2">Urgent GP/hospital review</td><td>1</td><td>0</td><td>0</td><td>1</td><td>n/a</td><td>n/a</td><td>n/a</td><td>n/a</td></tr>
<tr><td colspan="2">Reviewed risk benefit and decided not to start</td><td>2</td><td>0</td><td>0</td><td>2</td><td>n/a</td><td>n/a</td><td>n/a</td><td>n/a</td></tr>
<tr><td colspan="10">Footnote</td></tr>
</table>

*reduced by 3 (ml/min/1.73m^2) and discontinued
